# Thinking One Step Ahead: Strategies to Strengthen Epidemiological Data for Use in Risk Assessment

**DOI:** 10.1289/ehp.122-A311

**Published:** 2014-11-01

**Authors:** Carrie Arnold

**Affiliations:** Carrie Arnold is a freelance science writer living in Virginia. Her work has appeared in *Scientific American*, *Discover*, *New Scientist*, *Smithsonian*, and more.

Risk assessment is a cornerstone of environmental health research and policy making.^1^ A commentary in this issue of *EHP* presents a set of recommendations and guidelines to help researchers more effectively characterize uncertainty in epidemiological findings.^2^ Not only will this provide more transparency for the science itself, says coauthor Jennifer Pierson, a scientific program manager at the ILSI Health and Environmental Sciences Institute, it should also lead to more sound policies when those findings are integrated into risk assessments.

**Figure:**
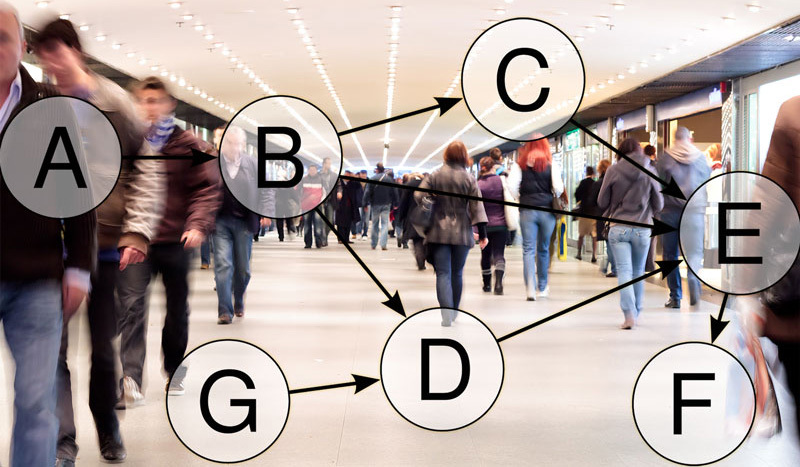
Directed-acyclic graphs (DAGs) can be an effective way to visualize relationships between the variables in a study. © Joseph Tart; Tomislav Pinter/Shutterstock

“Risk assessment is nothing magical; it’s a process to guide decision making. As with any kind of scientific question, it’s important to know how certain we are of our data,” says Thomas Burke, director of the Johns Hopkins Risk Science and Public Policy Institute, who was not involved with the commentary. “We can’t ever fully eliminate uncertainty, but we can describe it and put bounds around it with statistics.”

Experimental data have traditionally formed the basis for most human health risk assessments, but increasingly regulators are recognizing the value of epidemiological data for this purpose. “Different types of studies, like toxicology studies in animals and epidemiological studies in humans, can help compensate for each other’s inherent weaknesses,” says Michael Dourson, director of Toxicology Excellence for Risk Assessment, a public health organization located in Cincinnati, Ohio. Dourson was not involved with the commentary.

In October 2012 Pierson and colleagues convened a workshop with more than 30 environmental health researchers to develop recommendations for characterizing uncertainty in epidemiological studies. Their recommendations and guidelines form the basis of the new commentary.

To Pierson, one of the most important first steps is to get study authors out of their respective silos. “Oftentimes, the epidemiologists don’t work with the toxicologists, who don’t work with the risk assessors,” she says. By working together from the earliest planning stages of a study, researchers can pool their knowledge to limit uncertainty through study design, rather than scrambling to fix problems at the end.

Addressing uncertainty is critical when writing up results so that policy makers can factor it into their appraisal of the literature. Pierson and colleagues recommend that investigators assess and comment on the uncertainty in their findings using a tiered system developed by the National Research Council. This system enables policy makers to rate the quality of epidemiological data and how well study findings can be generalized to larger populations. This further allows them to weigh the uncertainties from different studies based on the quality of research, creating more accurate and nuanced risk assessments. For authors, applying the system to their own work can point to areas where uncertainty can benefit from further analysis.^2^

Validation studies and sensitivity analyses of epidemiological data, combined with a better understanding and disclosure of the sources of uncertainty, can help authors explore such areas. These methods can transform the discussion of uncertainty from its usual qualitative form^3^ into a quantitative measurement. This allows scientists to clearly communicate their results and accompanying uncertainties in the numbers-driven language of policy makers.^2^

“You need to communicate what you’ve done, and you’ve got to be able to state your results in a way that managers can get their head around,” Dourson says.

The authors of the commentary recommend several more techniques to more clearly and accurately present data. Among others, they suggest the use of directed-acyclic graphs as a way to visualize the sometimes complex relationships among confounders. They also emphasize the need to distinguish between correlation and causation in describing study results, to ensure scientists and policy makers don’t draw incorrect conclusions about risk.
